# High Efficiency Acetylcholinesterase Immobilization on DNA Aptamer Modified Surfaces

**DOI:** 10.3390/molecules19044986

**Published:** 2014-04-21

**Authors:** Orada Chumphukam, Thao T. Le, Anthony E. G. Cass

**Affiliations:** Department of Chemistry, Imperial College, London SW7 2AZ, UK

**Keywords:** aptamers, acetylcholinesterase, immobilization

## Abstract

We report here the *in vitro* selection of DNA aptamers for electric eel acetylcholinesterase (AChE). One selected aptamer sequence (R15/19) has a high affinity towards the enzyme (K_d_ = 157 ± 42 pM). Characterization of the aptamer showed its binding is not affected by low ionic strength (~20 mM), however significant reduction in affinity occurred at high ionic strength (~1.2 M). In addition, this aptamer does not inhibit the catalytic activity of AChE that we exploit through immobilization of the DNA on a streptavidin-coated surface. Subsequent immobilization of AChE by the aptamer results in a 4-fold higher catalytic activity when compared to adsorption directly on to plastic.

## 1. Introduction

*In vitro* selection of nucleic acid aptamers has become increasingly used to create new tailored molecules of interest for a variety of applications. Aptamers have been widely used in biosensors as well as therapeutics [1,2]. Aptamers can be selected to bind a target with a high level of discrimination over similar molecular architectures [3]. It has been shown that aptamers can be selected to bind to different regions of their targets, especially with large and structurally complex targets such as proteins. Examples include thrombin where two aptamers bind at distinctively different sites: the exosite-I (heparin binding site) [4] and the exosite-II (fibrinogen binding site) [5]. A second example is streptavidin, where some aptamers have been shown to bind in the biotin binding pocket [6] whilst others bind elsewhere on the molecule [7].

Enzyme immobilization is widely used in diagnostic assays and biosensors [8,9] as well as in bioreactors [10,11]. Traditionally enzyme are attached to surfaces through either adsorption [12] or covalent binding [13], both of which often result in substantial loss of activity and hence function as even small changes in structure can have a major impact on function [10]. Efforts to avoid this involve site-directed immobilization, commonly by the addition of affinity tags via recombinant DNA methods, which has become popular due to its one-step attachment strategy that immobilizes the enzyme molecules in a site-directed and oriented fashion and facilitates substrate access [14]. Such affinity tags include Biotin, His-tag, the FLAG octapeptide, Strep-II, glutathione S-transferase (GST), maltose binding protein (MBP), and heavy chain protein-C (HPC) which have been shown to be able to efficiently hold the enzymes without significantly disturbing their function [15,16,17,18]. However, these all suffer from the disadvantage that the enzymes to be immobilized have to undergo either chemical or genetic modification [19,20]. An alternative strategy is to use specific recognition of part of the protein by a surface bound reagent. In this respect, protein A has been used for site-directed immobilization of antibodies, especially in immunoassays. However as surface bound proteins are easily denatured, this simply shifts the problem from one protein to another [21]. Nucleic acid aptamers have been proven to be comparable to antibodies as recognition molecules and are used for analytical and diagnostic purposes [2], as well as in therapeutic applications [1,22]. There are established chemistries for DNA immobilization on a variety of surfaces and immobilized aptamers retain their binding capacity over a wide range of conditions [23,24,25]. This means that aptamers can be used for high affinity immobilization with the added advantage of easy reversibility, thermal or solvent induced denaturation of nucleic acids is completely reversible when initial conditions are restored. Robust and easy immobilization of nucleic acids has been demonstrated through the rich history of DNA microarrays and DNA biosensors [26,27,28,29]. For example, Gold *et al.* have described the use of aptamer microarrays for multiplexed protein analysis and applied this to identify biomarkers in proteomic populations [30,31]. In terms of the aptamer behavior in a microarray format, it was reported that they retained specificity for their targets even in the presence of high concentration of other proteins [32,33], suggesting that aptamers can be used for immobilization of enzymes from a complex matrix. This has the advantage of not requiring prior purification of the enzyme. 

**Figure 1:**
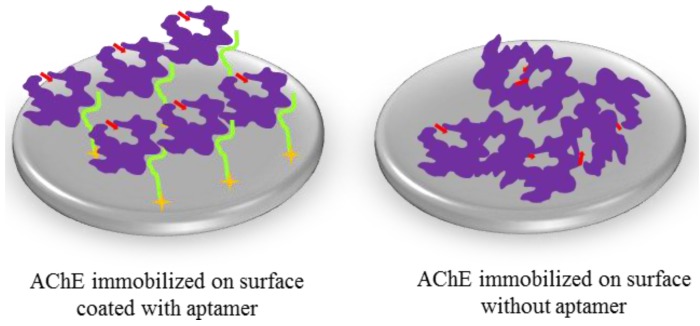
Site-directed immobilization of AChE mediated through aptamer binding versus adsorption of AChE to a polystyrene microtiter plate. The former allows specific orientation and well-controlled immobilization of AChE whilst the latter results in AChE immobilized in random orientations. The purple represents unmodified AChE molecules whilst the light green indicates the AChE-binding aptamer and the gold stars show the attachment chemistry of the aptamer molecules to the surface. The red arrows show AChE’s substrate channel. In our experiment, the aptamer was immobilized on a streptavidin-coated surface through biotin at its 5′- or 3′-end. Biotin incorporation is straightforward to implement during the synthesis of the aptamer. Alternatively the aptamer can be attached to gold surfaces through a 5′- or 3′-thiol end, a method particularly well suited to sensing applications.

In this paper, we present the use of an aptamer as a surface bound immobilization reagent. This aptamer binds AChE without interfering with or blocking the active site(s) of its target and therefore it has the potential to be used for high efficiency site-directed immobilization of the native enzymes as illustrated in Figure 1. 

## 2. Results and Discussion

### 2.1. Sequence Analysis and Binding Characterization

**Figure 2:**
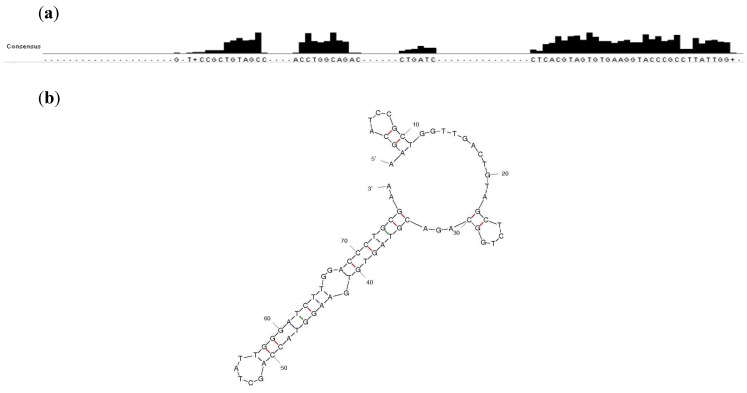
(**a**) Common motifs shared between the 47 clones of the enriched pool at round 15. Alignment was performed without including the primer regions. Sequence analysis was carried out using ClustalW2 [34]. (**b**) A predicted structure of R15/19 by Mfold [35].

The sequenced population of round 15 showed both common motifs shared between sequences as well as identical sequences (Figure 2). Sequence R15/19 AAGCATCCGCTGGTTGAC TGTAGCTCTG GCAGACGTAG TGTGAAGGTA CCAGCTATTGG GATCTTGGACCCTGCGAA was found in 19 out of 47 sequences (see Supplementary Figure S1). Sequence R15/C19 was chosen for further characterization.

This aptamer was selected to bind AChE with 77 nucleotides in total, but a number of nucleotides in this sequence may not be involved in binding to its target. It would be useful to minimize the sequence by excluding those nucleotides that have no involvement in the binding and truncation of this aptamer, results of which will be reported in another publication.

### 2.2. AChE Enzyme Activity

AChE is a very high turnover rate hydrolase where the catalytic site contains serine, histidine and glutamic acid residues instead of the aspartic acid residues found in most serine hydrolases [36]. A theory explaining the high catalytic efficiency is that anionic groups dominate the surface charge and a high net negative charge on the AChE active site is a driving force for the rapid association of cationic substrates [37]. Measurement of AChE activity in the presence of the R15/19 aptamer (negatively charged at the buffer condition), showed no significant change from its absence (see Supplementary Figure S3). This is consistent with the aptamer not binding to the substrate access region, which is known to be negatively charged. 

**Figure 3:**
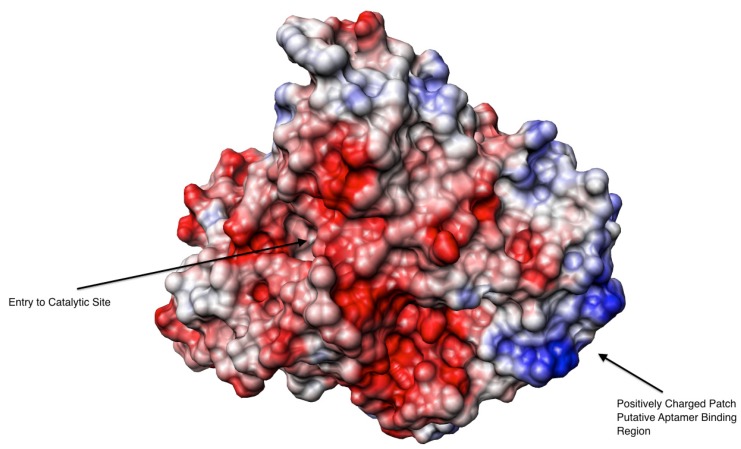
Columbic surface of an electric eel AChE tetramer created using Chimera software. The red color presents negative charge whilst the blue is for positive charge. The original structural data obtained from the X-ray diffraction with a resolution of 4.5 Å [38].

From the X-ray structure published by Bourne *et al*. [38], we observed AChE’s surface charge to identify plausible locations of aptamer binding. The columbic surface of AChE was visualized using Chimera (Figure 3). As expected, the AChE surface charges were not evenly distributed; revealing negatively charged patches surrounding the enzyme’s active site as proposed by Noltle *et al.* [37] based on enzyme kinetic studies and positively charged patches on the opposite side of the molecule. It is worth noting that the nucleic acid aptamer in the binding buffer condition (pH 7.4) is negatively charged and through electrostatic interactions the aptamer could favor binding to the positively charge areas. If the aptamer binds a positively charged patch of AChE, which is distant from the active site, it would not affect the enzyme’s activity through occupying the active site nor preventing substrate access.

### 2.3. Binding Affinity of the Aptamer to AChE

**Figure 4:**
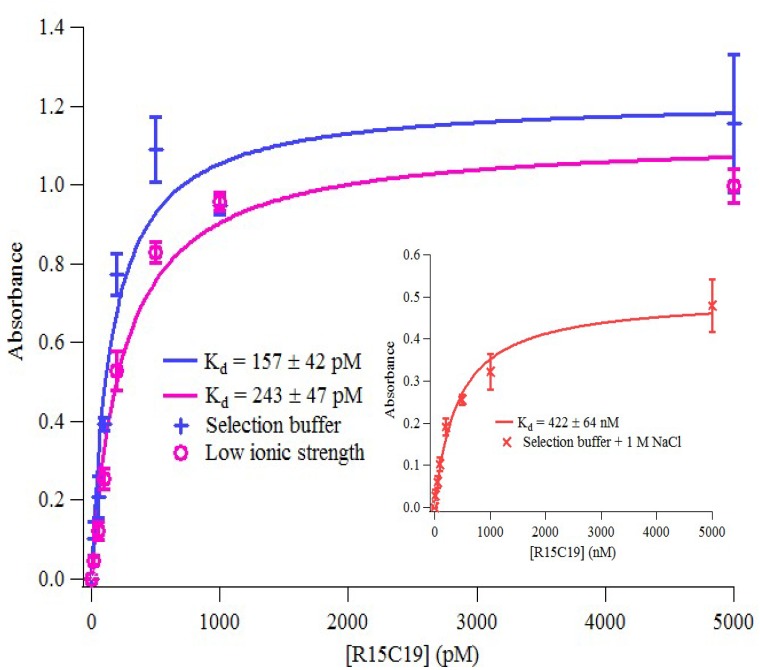
Binding of R15/19 to AChE under different conditions. The binding data were obtained from ELONA and were fitted into a hyperbolic equation (Langmuir model) using Igor Pro (WaveMetrics).

Binding of R15/C19 to AChE was measured using Enzyme Linked Oligonucleotide Assay (ELONA). The results show the aptamer binds AChE with high affinity (K_d_ = 157 ± 42 pM) whilst no binding to BSA or streptavidin was detected (see Supplementary Figure S4). In addition, binding of the aptamer to AChE under different buffer conditions were also investigated. Table 1 shows dissociation constants obtained from the binding curves in Figure 4.

**Table 1:** Dissociation constants of the aptamer R15/19 binding to AChE in different binding buffers. The K_d_ values were obtained using ELONA.

Binding buffer	Ionic strength (M)	K_d_ (pM)
Selection buffer (8 mM Na_2_HPO_4_, 2 mM NaH_2_PO_4_, 2.7 mM KCl, 137 mM NaCl, 4 mM MgCl_2_, pH 7.4)	~0.15	157 ± 42
Low ionic strength buffer (8 mM Na_2_HPO_4_, 2 mM NaH_2_PO_4_, 2.7 mM KCl, 4 mM MgCl_2_, pH 7.4)	~0.02	243 ± 47
High ionic strength buffer (8 mM Na_2_HPO_4_, 2 mM NaH_2_PO_4_, 2.7 mM KCl, 1,137 mM NaCl, 4 mM MgCl_2_, pH 7.4)	~1.2	(422 ± 69) × 103

As shown in Table 1, binding affinity of the aptamer to AChE is not markedly affected by reducing the ionic strength of the buffer, from ~150 mM to ~20 mM. However, under high ionic strength (~1.2 M), it showed a significant loss (over 3 orders of magnitude) in binding affinity. This could be due to the screening of the charges on the phosphate backbone of the aptamer as well as on the target, a phenomenon that was previously reported in other aptamer-target binding studies [39,40].

### 2.4. Immobilization of AChE Using R15/19

**Figure 5:**
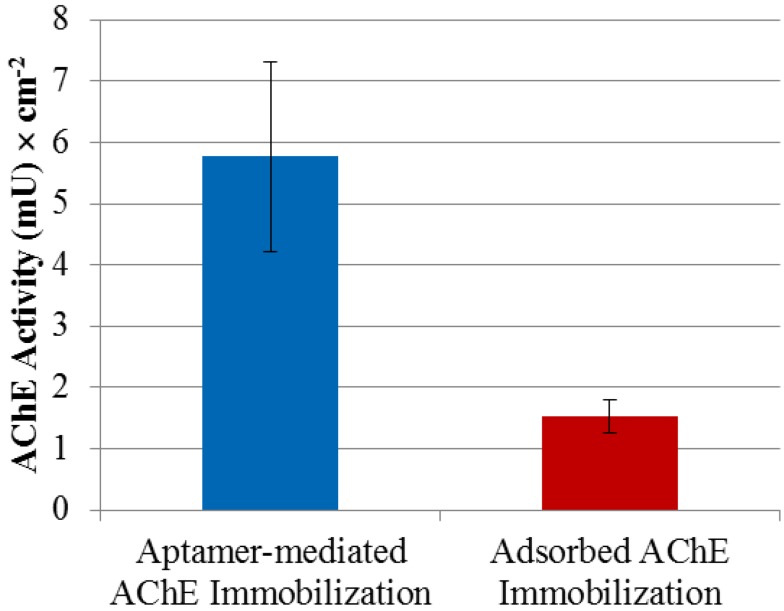
R15/19 as an immobilization mediator for AChE. Two immobilization methods for AChE were tested: adsorption of AChE to a polystyrene microtiter plate and affinity attachment by the aptamer-coated microtiter plate. The aptamer was immobilized on streptavidin-coated surface through biotin at its 3′-end. Negative control using polystyrene surface of a microtitier plate coated with streptavidin and blocked with BSA showed no significant amounts AChE activity and were used as background subtraction for the aptamer-mediated AChE immobilization data.

As shown earlier, the aptamer binds AChE with high affinity (K_d_ = 157 ± 42 pM) and without interfering with or blocking the enzyme’s substrate access, which makes the aptamer an ideal surface-bound reagent for site-directed immobilization of AChE for sensing platforms. Immobilization of AChE in a site-directed manner was achieved by first modifying the surface with streptavidin in order to subsequently assemble the R15/19 aptamer, which then captured AChE. Figure 5 compares the activity of AChE immobilized via aptamer capture with that from adsorption.

Figure 5 shows a 4-fold improvement in the activity of the immobilized enzyme in the former case compared to the adsorption method and is consistent with the observation that the aptamer does not inhibit AChE activity. There are a number of factors that cause loss of catalytic activity when enzymes are immobilized; these include surface induced denaturation and aggregation as well as steric hindrance of access by substrate to the active site. In the case of aptamer mediated immobilization it is possible that the absence of direct interaction between the protein and surface reduces the former whilst the flexible nature of the ssDNA chain linking the two allows for good substrate accessibility. That the aptamer could play this role well is due to a number of reasons. Firstly the binding affinity is comparable to that of the affinity tags for proteins that have been used widely in immobilization and affinity chromatography. Secondly, using this aptamer as an immobilization reagent does not require any additional modification of AChE. In addition, aptamers’ reversible denaturation, which is well demonstrated in DNA microarrays, makes this immobilization approach valuable in sensing devices, where the enzyme attached on the aptamer-mediated surface can be easily removed and replaced with the fresh enzyme. This property is particularly useful with labile enzymes. Moreover the method is easy to apply and could also be used for protein patterning when based on an underlying DNA template pattern. An alternative approach could be to use an antibody instead of an aptamer and this has indeed been described for other enzymes [41]. However there are compelling cost reasons to choose aptamers over antibodies. A commercial monoclonal antibody against AChE costs around £250 for 100 µg (equal to ~ £1 for 5 pmol). In contrast commercial oligonucleotide synthesis typical yields 5,000 pmol for £1. This is direct consequence of the low cost of oligonucleotide synthesis and the 10-fold lower molecular weight of the aptamer. A final point of comparison is that aptamers can be reversibly denatured on surfaces, unlike antibodies, which show irreversible loss of function when denatured.

## 3. Experimental Section

### 3.1. Selection of the Aptamer

A DNA library with a random region containing 41 nucleotides (AAGCATCCGCTGGTTGAC–N41-GATCTTGGACCCTGCGAA) was used. One nmol of DNA was used in the first round and about 200-280 pmol of DNA in subsequent selection rounds. The pool was incubated with AChE in selection buffer (8 mM Na_2_HPO_4_, 2 mM NaH_2_PO_4_, 2.7 mM KCl, 137 mM NaCl, 4 mM MgCl_2_, pH 7.4), in which AChE was decreased 100 to 5 pmol as the selection progressed. Nitrocellulose membrane (0.45 µm HAWP01300 Millipore) filtration was used to separate AChE bound from unbound molecules. A total of 15 selection rounds were performed. Along with reducing the ratio of the target protein to DNA, increasing washing stringency regimes were applied as the selection progressed. The bound sequences were then eluted with boiling water and PCR amplified using a forward primer (AAGCATCCGCTGGTTGAC) and a 5′-biotin modified reverse primer (biotin-TTCGCAGGGTCCAAGATC). ssDNA was prepared using streptavidin-coated magnetic beads. The obtained ssDNA pool was suspended the selection buffer and then was filtered through nitrocellulose membrane prior to pool-target binding for every selection round. Selection enrichment for binding with its target (AChE) was monitored using ELONA (see Supplementary Figure S2). The enriched pool was then cloned and 47 individual colonies were picked for sequencing. Analysis of the sequencing data identified consensus regions was performed using ClustalW2 [34]. Candidate sequences were then chosen based on frequency in the sequenced population for further characterization.

### 3.2. ELONA Assays

K_d_ values were determined using an Enzyme Linked Oligonucleotide Assay (ELONA) [42]. This is similar to ELISA whereby the target (AChE) is adsorbed in the wells of a microtiter plate and biotinylated aptamer titrated in, allowed to bind and then the wells washed with buffer. The amount of captured aptamer is then determined by adding streptavidin-HRP and suitable substrates. Finally the absorbance at 450 nm is measured (see Supplement Figure S2). One hundred µL of 10 nM AChE solution in PBS was added to each well of 96-well polystyrene plates and incubated at 4 °C overnight. The AChE solutions in the wells were then discarded and then were washed 4 times with 150 µL of PBS supplemented with 0.05% (v/v) Tween 20 (PBST) to remove weakly bound molecules before being blocked with 1% (w/v) BSA. 100 µL of selection buffer solution (for background) or a 5′-biotinylated DNA in the selection buffer solution was then added to each well and incubated at room temperature for 2 h. The unbound DNA was removed by washing four times with 150 µL of the selection buffer supplemented with 0.05% (v/v) Tween 20. The bound DNA was determined by adding 100 µL of a streptavidin-HRP solution to each well. Streptavidin-HRP is bound to the DNA through biotin at the 5′-end of the DNA. Excessive HRP was removed by washing four times with 150 µL of the selection buffer supplemented with 0.05% (v/v) Tween 20 and 0.1% (w/v) BSA. The amount of HRP retained in each well corresponds to the amount of bound DNA. Quantification of the HRP, hence the DNA, was done by measuring HRP activity using TMB substrate. The DNA solutions had a concentration range of two orders of magnitude to allow establishment of a binding curve for calculation of K_d_ values.

### 3.3. AChE Activity Assays

For absorption immobilization of AChE, various concentrations of AChE (0–50 nM) were prepared in two different buffers, 10 mM phosphate buffer at pH 7.4 and 10 mM sodium acetate (NaOAc) buffer at pH 5.2 to determine the concentration of AChE and the pH for a highest activity of the enzyme on the microtiter polystyrene plate surface. One hundred µL of an AChE solution was added to a well and incubated at 4 °C overnight. The AChE-coated plate was then washed 4 times with 150 µL of PBST to remove weakly bound molecules before being blocked with 1% (w/v) BSA. The AChE activity was determined using the Ellman method [43].

For aptamer mediated immobilization of AChE, 100 µL of 50 nM streptavidin solution was added to each well of a microtiter plate and incubated at 4 °C overnight. The plate was then washed four times with 150 µL of PBST to remove weakly bound streptavidin molecules before being blocked with 1% (w/v) BSA. One hundred µL of 100 nM solution of the biotinylated aptamer was then added to a well whilst 100 µL of selection buffer only (for negative control/background subtraction) was added to another well of the streptavidin-coated plate. The plate was then washed four times with 150 µL of selection buffer. One hundred µL of an AChE solution was added to each and then incubated for 2 h. The plate was then washed four times with 150 µL of selection buffer supplemented with 0.05% (v/v) Tween 20 and 0.1% (w/v) BSA to remove unbound AChE. Various concentrations of the AChE ranging from 0 to 200 nM were investigated to identify the condition for a highest enzyme activity The AChE activity was determined using the Ellman method.

## 4. Conclusions

We describes a DNA aptamer that binds AChE with high affinity (K_d_ = 157 ± 42 pM). Binding affinity of the aptamer to AChE was not affected at low ionic strength (20 mM) but suffers significant loss (over three orders of magnitude) under high ionic strength (1.2 M) conditions. Enzyme activity assays showed that this aptamer binds AChE without interfering with or blocking the enzyme’s substrate access channel. The aptamer was then used in surface modification for enzyme immobilization and gives a substantial retention of activity (4-fold) of AChE compared to adsorption.

## Abbreviations

AChE: acetylcholinesterase
ELONA: enzyme-linked oligonucleotide assay
BSA: bovine serum albumin
PBST: phosphate buffer saline Tween

## Supplementary Materials

Supplementary materials can be accessed at: http://www.mdpi.com/1420-3049/19/4/4986/s1.
