# Supplementary material for: High Efficiency Acetylcholinesterase Immobilization on DNA Aptamer Modified Surfaces
Source: Molecules. 2014 Apr 21;19(4):4986–96. doi: 10.3390/molecules19044986 (PMC6271157; doi:10.3390/molecules19044986)

Supplementary Materials

Figure S1. Sequence analysis of the enriched library at the round 15th of the selection. Alignment was performed without the priming regions. Sequences R15/19 was found in 19 out of 47 clones. Sequence analysis was carried out using Clustal W2 (http://www.ebi.ac.uk).

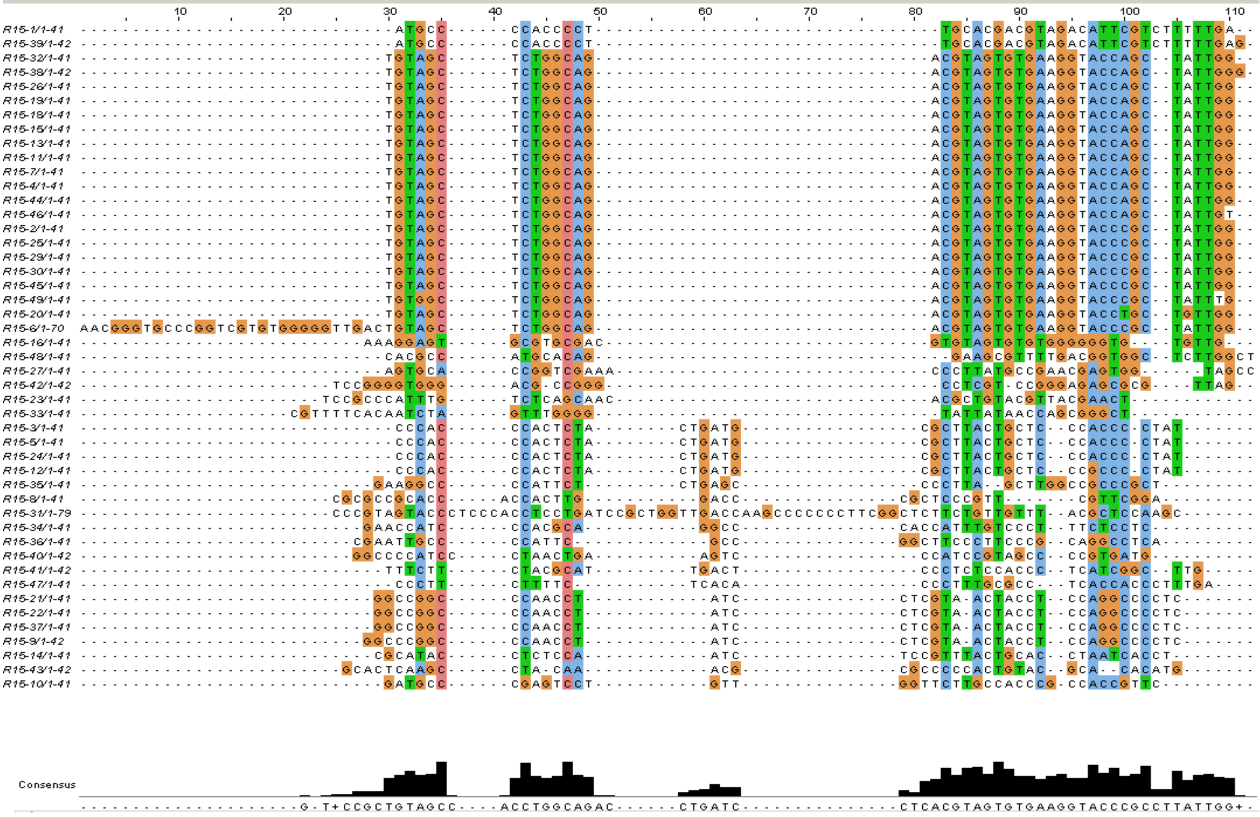

Figure S2. ELONA binding measurements of the ssDNA pools toward AChE over the course of selection. The 96-well plate was coated with 10 nM AChE and incubated with 20 nM ssDNA of the starting pool as well as the enriched pools. The cartoon illustrates the binding measurement scheme.

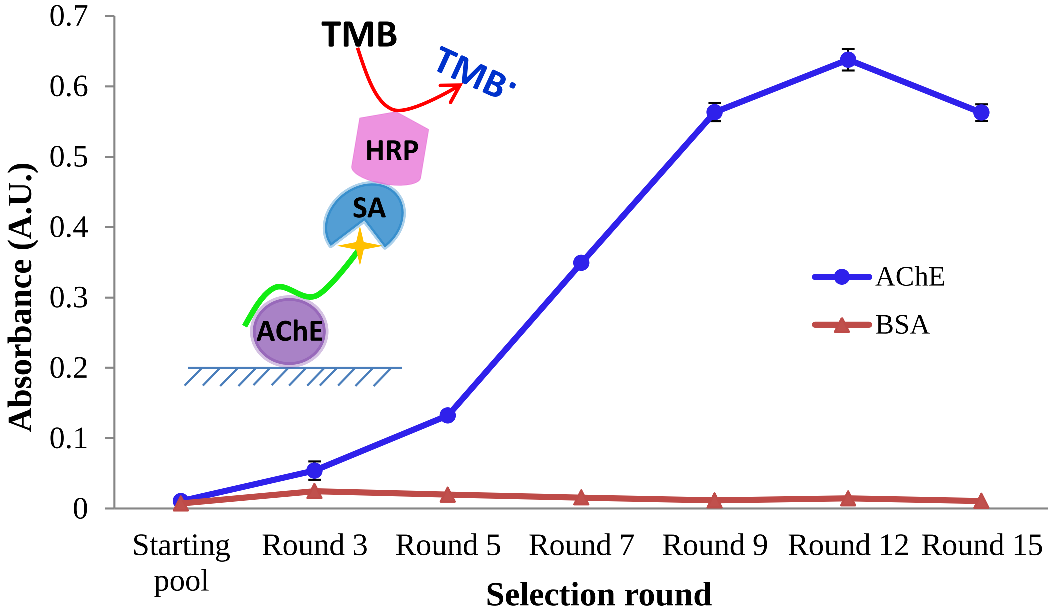

Figure S3. Relative activity of AChE in the absence (0 nM) and the presence of aptamer R15/19 at 40 nmM, 100 nM and 200 nM. The AChE activity was determined using Ellman method.

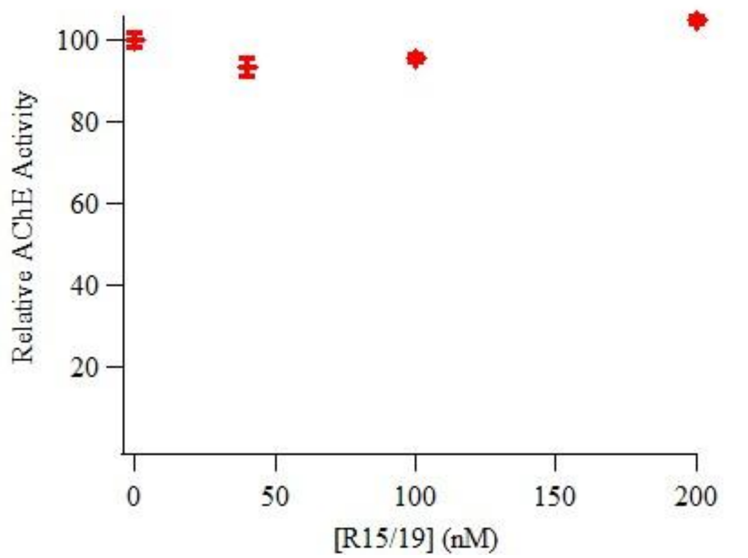

Figure S4. Binding of the aptamer R15/19 to AChE, Streptavidin and BSA.

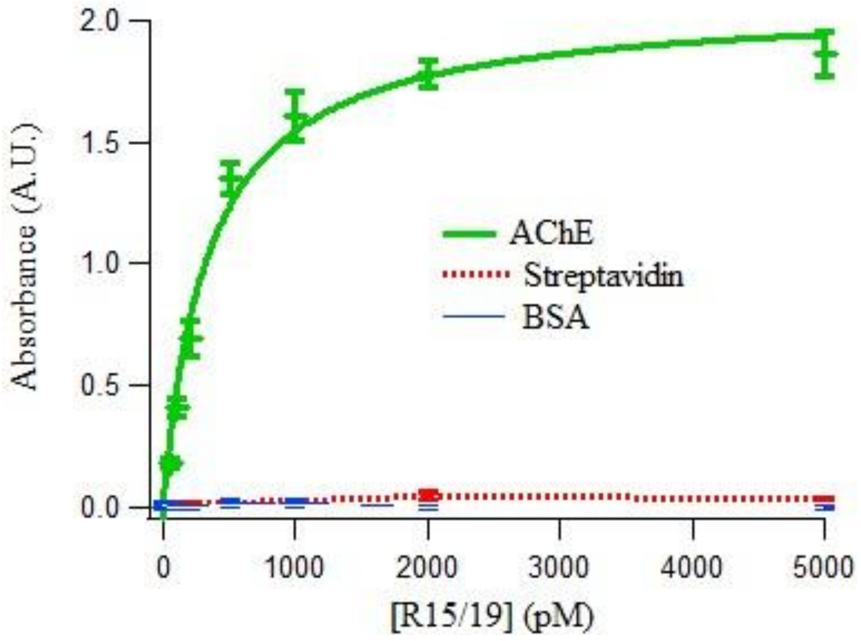

Supplement: Supplementary file 1 [file molecules-19-04986-s001.pdf]
